# Supplementary material for: Distinct growth regimes of α-synuclein amyloid elongation
Source: Biophys J. 2023 May 11;122(12):2556–63. doi: 10.1016/j.bpj.2023.05.009 (PMC10323017; doi:10.1016/j.bpj.2023.05.009)
Supplement: Document S1. Figures S1–S6 [file mmc1.pdf]

Biophysical Journal, Volume *122*

Supplemental information

Distinct growth regimes of α-synuclein amyloid elongation

Istvan Horvath, Hannah Welte, Jeremy D. Schmit, Michael Kovermann, and Pernilla Wittung-Stafshede

Supplemental Information

Distinct growth regimes of α-synuclein amyloid elongation

Istvan Horvath, Hannah Welte, Jeremy D. Schmit, Michael Kovermann, and Pernilla Wittung-Stafshede

Content:

Figures S1-S6 pages 2-7

Theory pages 8-11

A

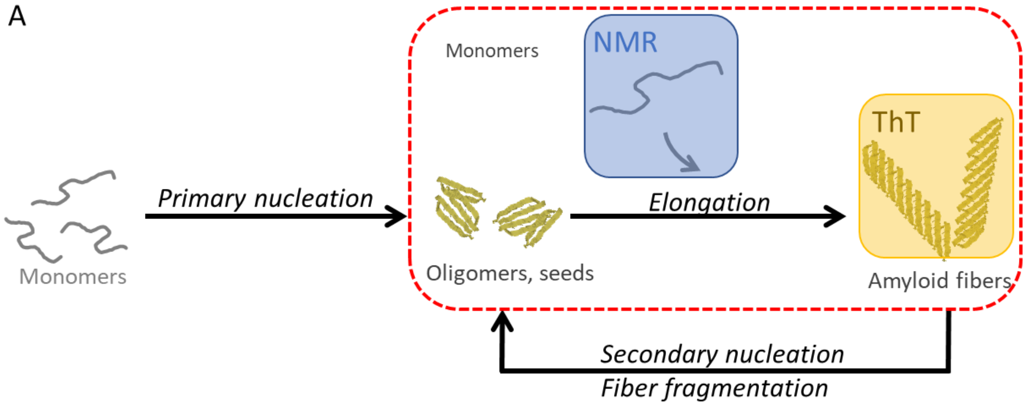

B

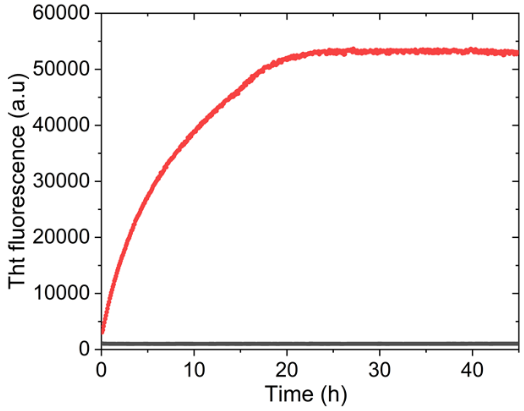

Figure S1

(A) Amyloid formation mechanism including primary and secondary processes highlighting the methods (NMR, ThT) and pathways (red box; elongation) used in this study. (B) Incubation of 100 µM monomers in the absence (black) and in the presence of 1 µM pre-formed amyloid fiber seeds (red) in quiescent conditions, showing lack of primary nucleation within the time window measured in the absence of seeds.

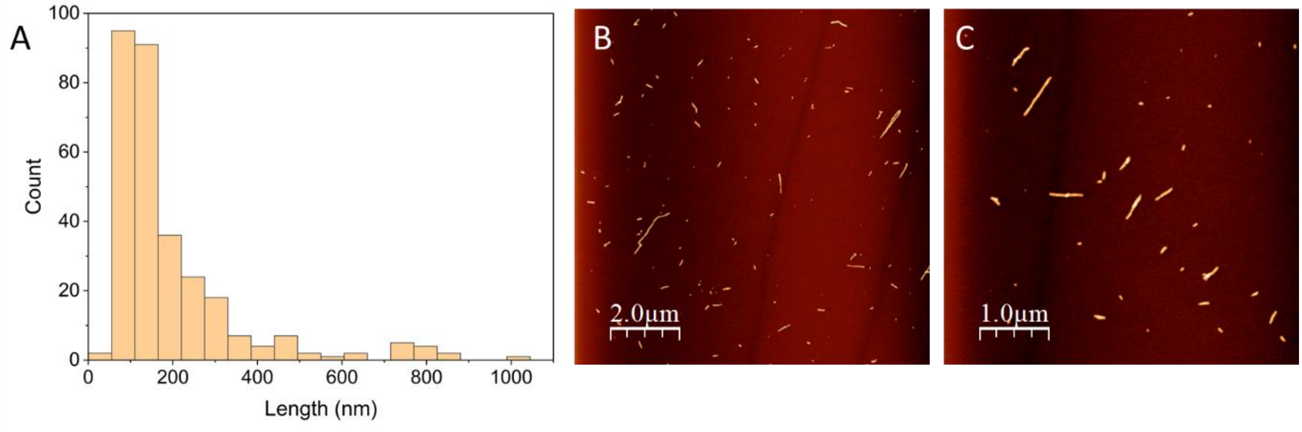

Figure S2

Size characterization of the used amyloid fiber seeds by AFM. Histogram of fiber lengths as determined from AFM images (mean length: 195 nm ±162 nm, median: 139 nm) (A), and two representative AFM images of the seeds, Z-range is set to 0-10 nm (B, C).

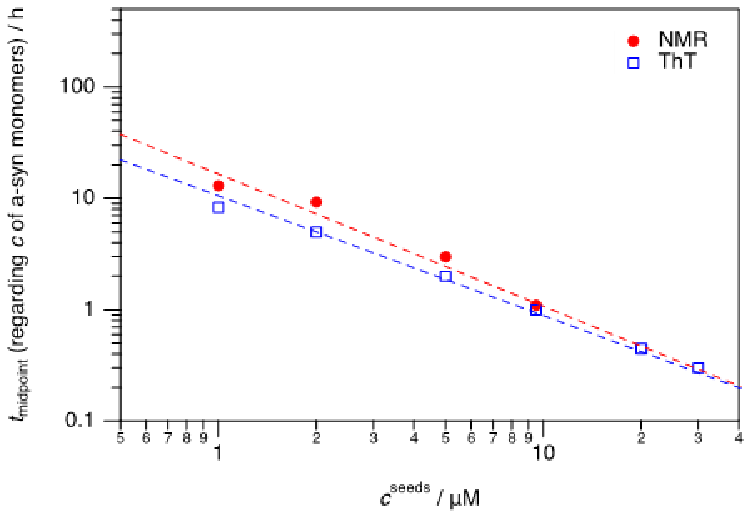

Figure S3

Double logarithmic plot of the midpoints of the seeded aggregation kinetics of aS monomers, $t_{midpoint}$, versus the concentration of pre-formed fiber amyloid seeds, c^{seeds}. The midpoints were determined from NMR spectroscopy (circles, red) and ThT fluorescence (rectangles, blue) experiments using 100 μM aS monomers, at T = 310 K (see **Table 1**). The dashed lines are linear fits (in the double logarithmic plot) to the data sets.

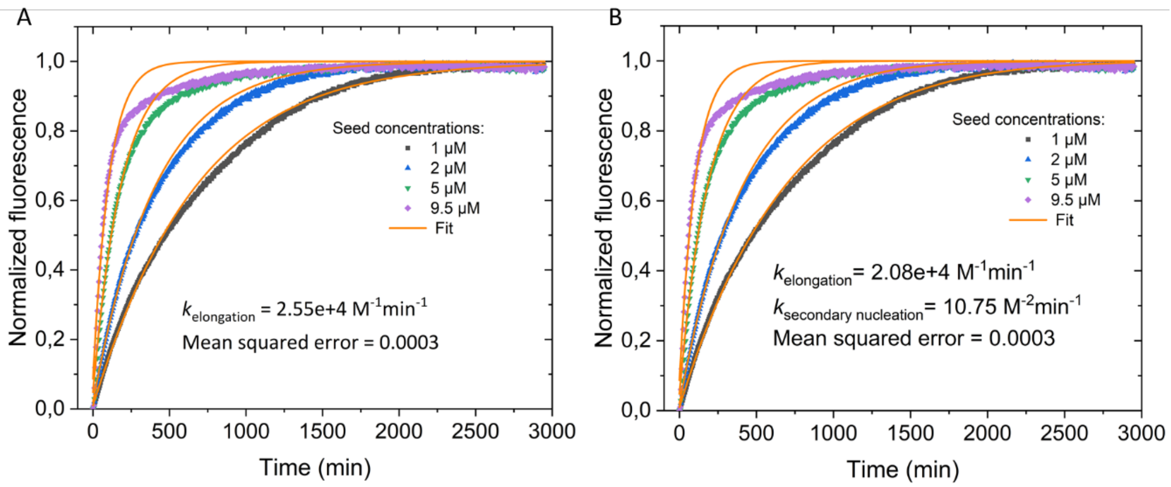

Figure S4

Global analysis by Amylofit of seeded aggregation of 100 μM aS monomers probed by ThT fluorescence at varying amyloid seed concentration. Two different fitting approaches were pursued here: elongation only (A) and elongation combined with secondary nucleation (B). Fitted curves are highlighted in orange in (A) and (B). For each condition, four replicates of experimental data were used for fitting; the plotted experimental curves are the averages.

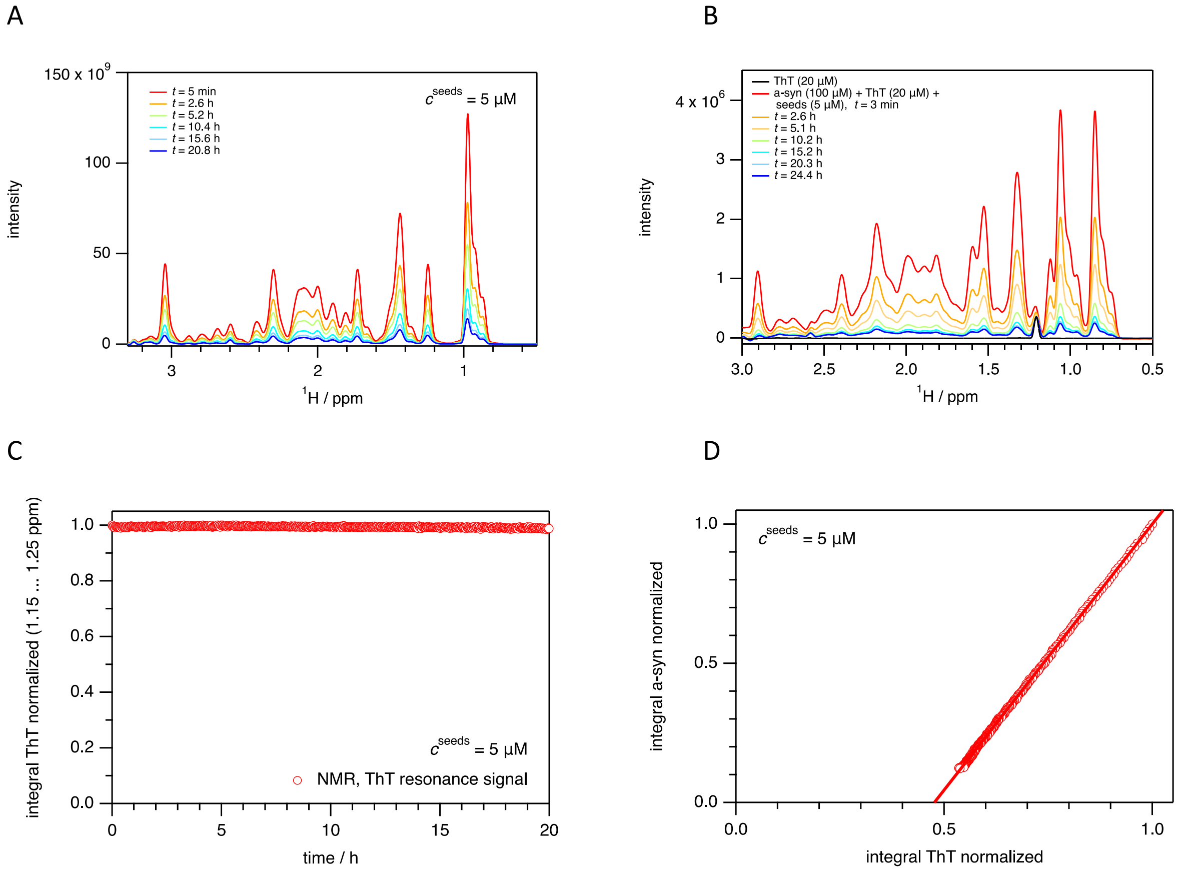

Figure S5

NMR spectroscopic data for aS and ThT at different conditions, at T = 310 K. (A) Real time 1D ^{1}H NMR data following the aggregation process of aS monomers at a concentration of 100 μM in presence of 5 μM amyloid seeds, in absence of ThT. The color coding refers to distinct time points within the series of acquired spectra. Corresponding data are shown in **Figure 1B**. (B) Real time 1D ^{1}H NMR data following the aggregation process of aS monomers at a concentration of 100 μM in presence of 5 μM seeds and in presence of 20 μM ThT. The spectrum colored in black represents the chemical shift observed for ThT. Note that aS has been ^{13}C isotopically enriched here enabling the separate analysis of the resonance signal of ThT within the series of acquired 1D ^{1}H NMR spectra. (C) The integral originating from the resonance signal of ThT was also followed in real time for a sample composed of 20 μM ThT and 5 μM seeds only (control; no change in integral reporting on ThT). (D) NMR data analysis of the aggregation process of aS monomers was conducted by determining the integral of resonance signals arising from the aS only sample (y-axis) and the sample comprising aS and ThT (x-axis). Both experimental set ups give the same numerical parameters for A_{slow}, A_{fast}, k_{slow} and k_{fast}. The continuous line colored in red represents this correlation leading to the conclusion that ThT does not affect aS elongation kinetics.

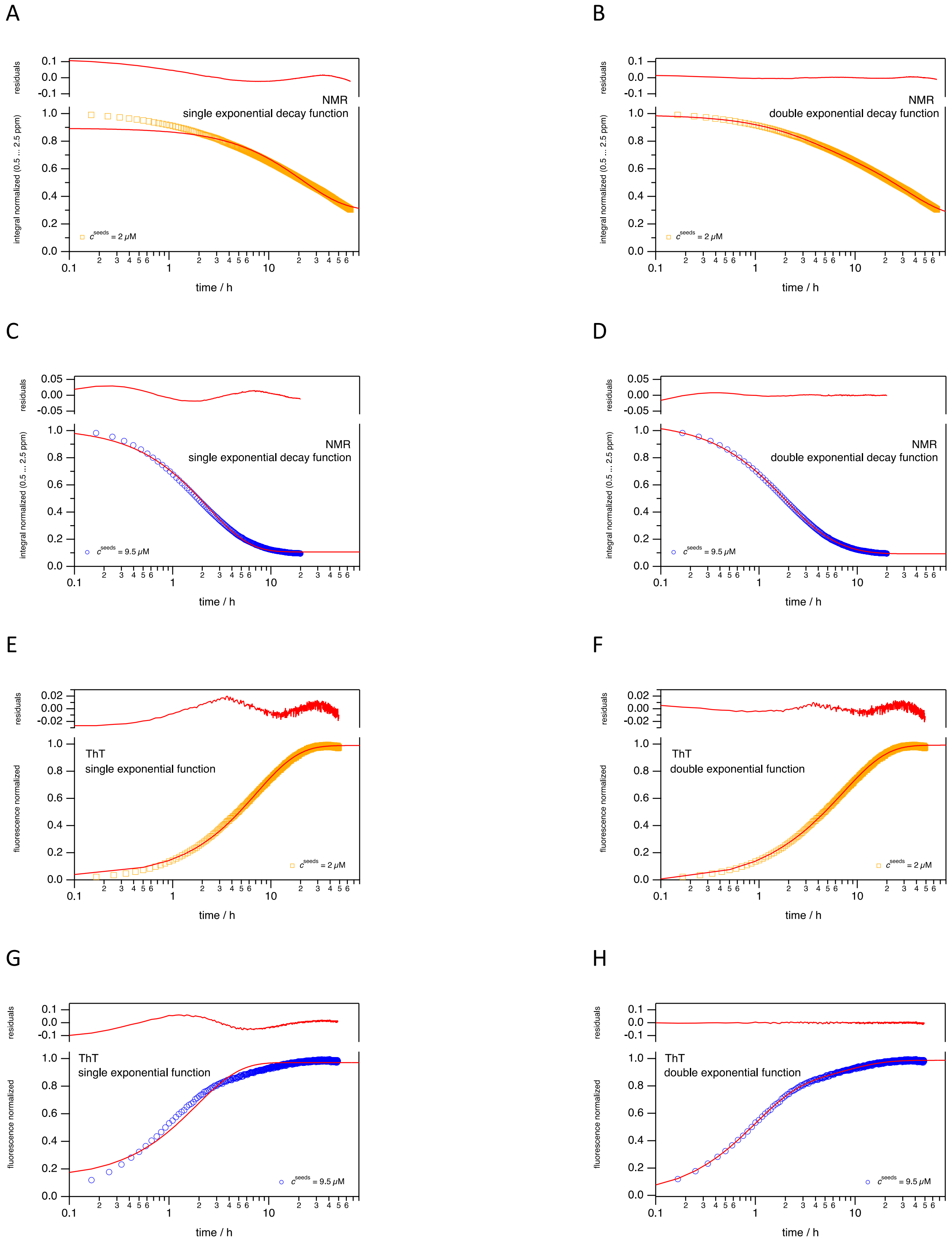

Figure S6

Fitting of the experimental data with exponential functions comprising one (A, C, E, G) or two kinetic phases (B, D, F, H). Fitting results are shown for data obtained by ThT fluorescence (E- H) and by NMR spectroscopy (A- D). The seeded aggregation of aS monomers was followed in presence of pre-formed fibers at a concentration of 2 μM (A, B, E, F) as of 9.5 μM (C, D, G, H). The progression of residuals depends significantly on the number of kinetic phases used in the fitting procedures. So bi-phasic fits giving significant improved match to experimental data compared to mono-phasic fits.

Theory

We considered three amyloid fiber elongation models to explain the biexponential behavior that we observed in our experiments. Our mathematical modelling is explained below:

1. Two pathways model

One possibility is that the two exponential regimes are indicative of different pathways by which monomers can attach to the end of a fibril. Simulations (1) have shown that monomer deposition can occur either by direct attachment from the solution or by first adhering to the side of the fibril and diffusing laterally to the end. The increase in fibril mass comes from the sum of the two pathways

$$\frac{dc_{fibril}}{dt} = J_{direct} + J_{side}$$

Where

$$J_{direct} = k_{direct} c_{sol} c_{end}$$
$$J_{side} = k_{side} \rho_{side} c_{end}$$

The direct pathway is proportional to the concentration of fibril ends c_{end}, the concentration of soluble protein c_{sol}, and a rate constant k_{direct}. Similarly, the side pathway will be proportional the concentration of protein bound to the sides of the fibril. However, because the concentration of binding sites per length of fibril is unknown, we have expressed the side concentration as a dimensionless density, ρ_{side}. This means that the rate constant for side pathway growth, k_{side}, has different units than k_{direct}.

It is an excellent approximation to treat c_{sol} and ρ_{side} in the pseudo-equilibrium limit. To see this, we can compare the molecular collision rate to the growth rate of the fibril. A random coil polypeptide chain has a radius of ~2.5 nm (2), which gives a Stokes-Einstein diffusion constant of $D = 10^2\ \mu m^2/s$. Inserting this value in the Smoluchowski formula gives $(4\pi D a c_{sol})^{-1} \cong 10^5/s$ as the collision rate, where we have a used a target radius of $a = 2$nm and the maximum concentration of $c_{sol} = 100\ \mu M$. To estimate the fibril elongation rate, we need to know the concentration of fibril ends. AFM measurements give an average length of 195 nm, which works out to about 830 molecules per seed using 0.47 nm per layer and 2 molecules per layer. Accounting for the fact that there are two growing ends per fibril gives $c_{end} = c_{seed}/415$. To estimate the initial elongation rate, we fit the first ten ThT measurements to the function $c_{sol} = (100\ \mu M)Exp[-k_{app}t]$ to find the apparent decay rate (**Theory Figure 1 top**).

The fitted rate constants show a linear trend with seed concentration $\frac{k_{app}}{c_{seed}} = 0.081/\mu M/hr$ (**Theory Figure 1 bottom**).

The initial fibril conversion rate is $\frac{dc_{fibril}}{dt} = (100\ \mu M)k_{app}$, which gives a conversion rate per end $\frac{(100\ \mu M)k_{app}}{c_{end}} = 0.93/s$. Because this is 5 orders of magnitude slower than the collision rate, the monomer distribution along the fiber surface will re-equilibrate between monomer addition events to the fiber ends. Therefore, no difference in monomer concentration along the fibers needs to be considered.

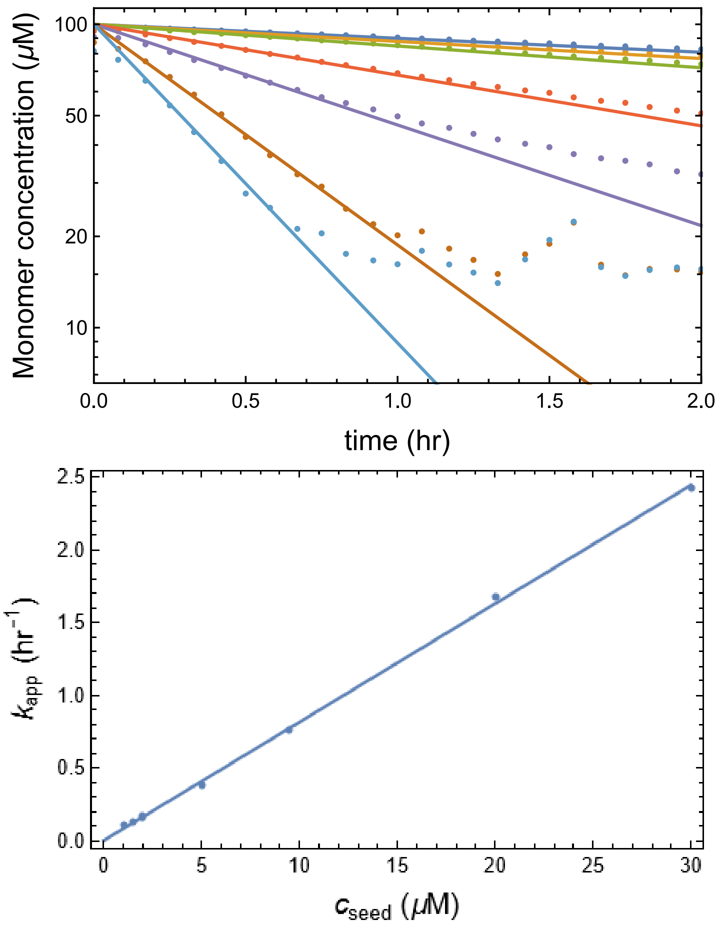

Theory Figure 1. ***Top:*** Fits of the initial fibril elongation rate to $c_{sol} = (100\ \mu M)Exp[-k_{app}t]$. ***Bottom:*** The apparent rate constants obtained from this fitting scale linearly with the seed concentration.

To find an expression for ρ_{side} we assume that there is a concentration c_{site} of binding sites on the side of fibrils, of which c_{bound} are bound to a monomer. The dissociation constant for the side binding sites is given by $k_d = c_{sol}(c_{site} - c_{bound})/c_{site}$, which can be rearranged to give $\rho_{side} = c_{bound}/c_{site} = c_{sol}/(k_d + c_{sol})$.
Inserting this into Equations [1-3], we have

$$\frac{dc_{fibril}}{dt} = c_{end}(k_{direct}c_{sol} + k_{side}\frac{c_{sol}}{k_d + c_{sol}})$$

It is instructive to look at two limits of this equation. If $c_{sol} \ll k_d$ we have

$$\frac{dc_{fibril}}{dt} \cong c_{end}(k_{direct}c_{sol} + k_{side}\frac{c_{sol}}{k_d})$$
$$\frac{dc_{fibril}}{dt} \cong c_{sol}c_{end}(k_{direct} + \frac{k_{side}}{k_d})$$
$$\frac{dc_{fibril}}{dt} \cong c_{sol}k_{app}$$

Where $k_{app} = c_{end}(k_{direct} + \frac{k_{side}}{k_d})$. Thus, at low concentration the two pathways merge to give a single effective conversion rate. In the opposite limit we have $c_{sol} \gg k_d$, which gives

$$\frac{dc_{fibril}}{dt} \cong c_{end}(k_{direct}c_{sol} + k_{side})$$

To get a "fast" elongation regime that is distinct from the slow elongation at low concentration it is necessary that $k_{side} \gg k_{direct}c_{sol}$. However, in this limit the fibril elongation rate is independent of the monomer concentration, which results in linear, not exponential, kinetics. Thus, this model does not support the observed biexponential kinetics.

2. Stop-and-go model

Another possible explanation for biexponential behavior is "stop and go" growth kinetics in which fibril ends switch between growing and arrested states. This could explain biexponential kinetics if all fibrils started in the growing state and gradually converge to a steady state population of arrested ends. This model predicts that the transition to the slow phase is time dependent (all systems will start fast and transition to the slow phase with a time dependence that is independent of the seed concentration), which contrasts to the "two pathways" model above which predicts a transition that depends on the monomer concentration. To evaluate this, we plot the fibril conversion rate per seed as a function of the monomer concentration (**Figure 3**, main manuscript; again, shown below as **Theory Figure 2**). Apart from the noisy behavior at long times in the low seed experiments, we see excellent data collapse, which supports the notion that the transition from fast to slow kinetics is a function of concentration, not time. The common break point between slow and fast regimes (around 20 μM) in **Theory Figure 2** corresponds to very different reaction times between the experiments shown (the midpoints of the seeded reactions span more than one order of magnitude (see **Figure 1A**, **Table 1** in main manuscript).

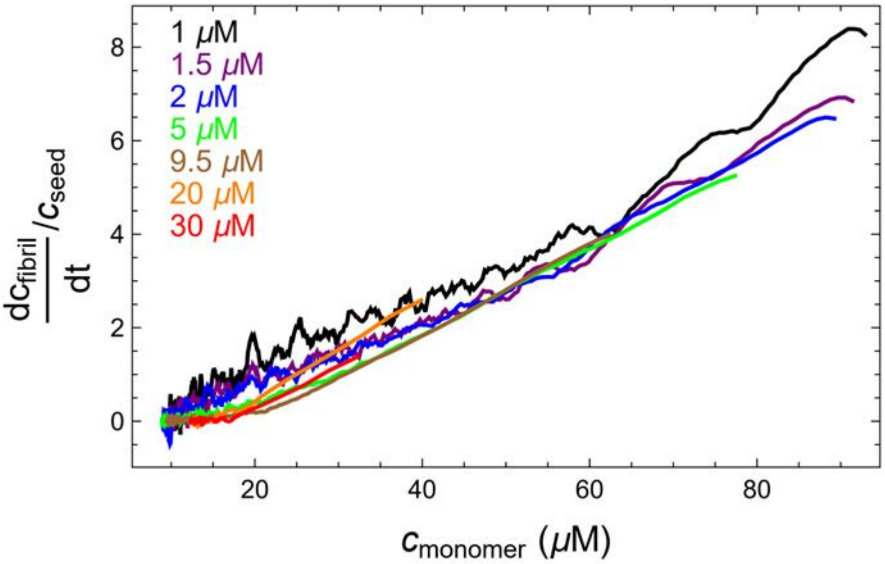

Theory Figure 2. Plot of fibril conversion rate per seed as a function of monomer concentration obtained from the ThT kinetic data (units: hr^{-1}). The curves have been smoothed by averaging over a window of ± 4 adjacent data points.

3. Disordered aggregation model.

With two pathways and stop-and-go disfavored, we turned to a third possible model, termed 'disordered aggregation model'. In **Theory Figure 2**, we see that the fibril elongation rate scales linearly with concentration when the monomer concentration is greater than about 20 μM. The conversion rates slow considerably at concentrations less than this and ultimately reach zero at the saturation monomer concentration near 10 μM. Two kinetic regimes with different monomer-dependence agree with the observed experimental biphasic behavior. We propose (see also main text discussion) that the slow growth regime observed in our experiments is consistent with the plateau

regime; the conformational search is too slow for the diffusion-limited regime to appear under these conditions. The fast growth regime would then be explained by a molecule deposition rate that overwhelms the conformational search resulting in disordered molecules incorporated within the fibril. The data in **Theory Fig 2**, is the ThT-fluorescence derived kinetics for which we have the largest data sets of (seed) concentrations. Although fewer kinetic traces, we also analyzed the NMR kinetics in the same way (**Theory Figure 3).** Although noisier, the same trend with two distinct regimes and data collapse (when plotted per seed) are indeed noted.

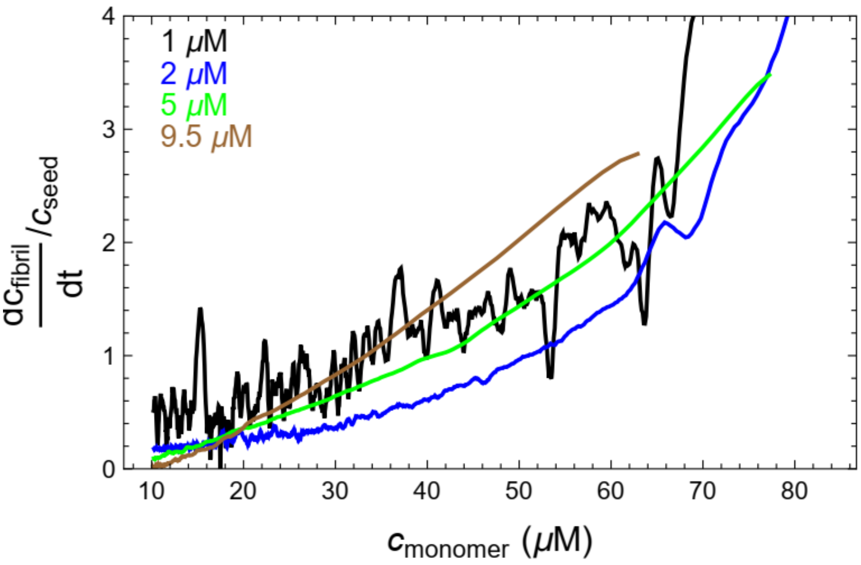

Theory Figure 3. Plot of fibril conversion rate per seed as a function of monomer concentration obtained from the NMR kinetic data (units: hr^{-1}). The curves have been smoothed by averaging over a window of ± 4 adjacent data points.

References

1. J. D. Schmit, Kinetic theory of amyloid fibril templating. *J Chem Phys* **138**, 185102 (2013).
2. J. E. Kohn *et al.*, Random-coil behavior and the dimensions of chemically unfolded proteins. *Proceedings of the National Academy of Sciences of the United States of America* **101**, 12491-12496 (2004).
