# Supplementary material for: Distinct growth regimes of α-synuclein amyloid elongation
Source: Biophys J. 2023 May 11;122(12):2556–63. doi: 10.1016/j.bpj.2023.05.009 (PMC10323017; doi:10.1016/j.bpj.2023.05.009)
Supplement: Document S2. Article plus supporting material [file mmc2.pdf]

Biophysical Journal
Article

Biophysical Society

Distinct growth regimes of α-synuclein amyloid elongation

Istvan Horvath,[1] Hannah Welte,[2] Jeremy D. Schmit,[3,*] Michael Kovermann,[2,*] and Pernilla Wittung-Stafshede[1,*]
[1]Department of Life Sciences, Chalmers University of Technology, Gothenburg, Sweden; [2]Department of Chemistry, University of Konstanz, Konstanz, Germany; and [3]Department of Physics, Kansas State University, Manhattan, Kansas

ABSTRACT Addition of amyloid seeds to aggregation-prone monomers allows for amyloid fiber growth (elongation) omitting slow nucleation. We here combine Thioflavin T fluorescence (probing formation of amyloids) and solution-state NMR spectroscopy (probing disappearance of monomers) to assess elongation kinetics of the amyloidogenic protein, α-synuclein, for which aggregation is linked to Parkinson's disease. We found that both spectroscopic detection methods give similar kinetic results, which can be fitted by applying double exponential decay functions. When the origin of the two-phase behavior was analyzed by mathematical modeling, parallel paths as well as stop-and-go behavior were excluded as possible explanations. Instead, supported by previous theory, the experimental elongation data reveal distinct kinetic regimes that depend on instantaneous monomer concentration. At low monomer concentrations (toward end of experiments), amyloid growth is limited by conformational changes resulting in β-strand alignments. At the higher monomer concentrations (initial time points of experiments), growth occurs rapidly by incorporating monomers that have not successfully completed the conformational search. The presence of a fast disordered elongation regime at high monomer concentrations agrees with coarse-grained simulations and theory but has not been detected experimentally before. Our results may be related to the wide range of amyloid folds observed.

SIGNIFICANCE Amyloid formation is a toxic process that underlies neurodegenerative diseases such as Parkinson's disease. To develop cures, mechanistic understanding of amyloid fiber assembly is needed. Amyloids grow longer in a process called elongation, which is believed to involve addition of monomers directly to the ends of an existing fiber. By combining two independent experimental methods, followed by rigorous data analysis, we demonstrate that elongation of α-synuclein amyloids involves distinct kinetic regimes. At high monomer concentration, we find rapid, disordered growth, whereas at low monomer concentration, growth involves ordered addition of monomers. The presence of a disordered growth regime, only noted in simulations before, may relate to variations in amyloid fold and stability.

INTRODUCTION

Amyloid fibrils are long polymers of monomeric protein units noncovalently assembled through β-strands in a cross-β structure (1). Many proteins can form amyloid fibrils at certain solvent conditions (1). In living organisms, amyloid fibrils can be functional (2), but most often, in humans, they are noted for their involvement in diseases (3–6). Amyloid formation of the protein α-synuclein (aS) is a hallmark of Parkinson's disease (PD), the second most common neurodegenerative disorder after Alzheimer's disease (7,8).

aS amyloids are the major content of pathological inclusions, Lewy bodies, found in the substantia nigra region in PD patient brains (9–11). Duplications, triplications, and point mutations in the aS gene are linked to familial PD cases, highlighting the importance of this protein (12). aS is an intrinsically disordered monomer in solution but adopts α-helical structure when interacting with lipid vesicles (13). Although not fully resolved, the function of aS appears to be related to synaptic vesicle release and trafficking (14–16).

Amyloid formation of aS and most other amyloidogenic proteins proceeds via at least two reaction steps: primary nucleation and elongation of fibrils (1,17). Additionally, amyloid formation can be catalyzed by secondary processes including secondary nucleation and fibril fragmentation (1,17,18) (illustrated in Fig. S1 *A*). For aS, primary

Submitted December 14, 2022, and accepted for publication May 8, 2023.
*Correspondence: schmit@phys.ksu.edu or michael.kovermann@uni-konstanz.de or pernilla.wittung@chalmers.se
Editor: Samrat Mukhopadhyay.
https://doi.org/10.1016/j.bpj.2023.05.009
© 2023 Biophysical Society.
This is an open access article under the CC BY license (http://creativecommons.org/licenses/by/4.0/).

processes dominate at physiological pH, whereas at lower pH, secondary processes become more important (19,20). In the presence of preformed amyloid fibrils (so-called seeds), the slow primary nucleation step of the process is bypassed. In this situation, monomers can rapidly attach to the preexisting fiber seeds and elongate those. Amyloid fibril seeding, and thus elongation, is believed to have importance in prion-like spreading of aS pathology in the brain (21). aS amyloid fiber elongation has been extensively investigated in vitro with, e.g., Thioflavin T (ThT) fluorescence (dye that emits when bound to amyloids (22)) and to some degree by other experimental approaches such as quartz crystal microbalance (23), surface plasmon resonance (24), as well as high-resolution fluorescence microscopy (25,26). Although microscopic analysis of individual fibrils showed elongation to exhibit a "stop-and-go" behavior (27), bulk experiments have mostly focused on the analysis of initial events. In the latter, initial rate constants are plotted against seed concentration, showing a linear correlation if only elongation is involved (18) or global fitting to analytical rate equations is applied (1,17).

To assess the molecular process of aS amyloid fiber elongation at physiological pH, we here combined two independent methods: ThT fluorescence, which probes amyloid formation, and one-dimensional proton solution state NMR spectroscopy, which probes disappearance of soluble monomers. Importantly, at these seeded conditions, there is no primary nucleation nor any secondary processes taking place. Surprisingly, kinetic profiles for aS elongation reactions at different amyloid fibril seed concentrations could be reliably fitted by using double exponential functions. The parameters (kinetic rate constants and amplitudes) were similar regardless of the method used for detection. To explain the observation of two kinetic phases, various mechanistic models were tested mathematically in which parallel pathways as well as the stop-and-go mechanism could not explain the experimental observations. Instead, we find that the experimental data is a consequence of differential elongation-rate dependence on instantaneous monomer concentration. In accord with coarse-grained simulations (28), we detect distinct kinetic regimes for α-synuclein fiber elongation here, including the elusive so-called "disordered aggregation" phase.

MATERIALS AND METHODS

Protein expression and purification

Wild-type aS protein was expressed in *E. coli* grown in LB (no isotopic labeling of aS) or M9 medium (containing ^{13}C glucose and ^{15}N ammonium chloride) and purified using anion exchange chromatography and gel filtration as previously reported (29). The purified protein aliquots were stored at −80°C. Before each experiment, gel filtration was performed to obtain homogeneous monomeric aS solution using a Superdex 75 10/300 (Cytiva, Uppsala, Sweden) column in TBS buffer (50 mM Tris, 150 mM NaCl, pH 7.6 at 25°C, Medicago, Uppsala, Sweden).

Preparation of aS preformed amyloid fibers (seeds)

100 μM of aS was incubated with agitation using glass beads at 37°C in TBS. Under these conditions the aggregation of aS is complete after less than 72 h. After 3 days of incubation, the aggregated protein was added to 250 μM fresh aS monomers so that the concentration of preaggregated protein is 5% of the monomeric. The mixture was incubated for 5 days at 37°C. Following the incubation, the sample was sonicated to obtain short fiber seeds, thus increasing the number of growing ends, and sonication was performed for 10 s using a probe sonicator (stepped microtip and Ultrasonic Processor Sonics Vibra-Cell; Sonics & Materials, Newtown, CT) running an amplitude of 20% and an alternating cycle of 5 s (on mode) and 5 s (off mode). The sonicated fibers were characterized by atomic force microscopy (Fig. S2). The sonicated fiber seed solutions were aliquoted, flash frozen in liquid nitrogen, and stored at −80°C until usage.

Seeded aggregation of monomeric aS followed by ThT fluorescence

Freshly gel-filtered aS monomers at 100 μM concentration in TBS were first mixed with ThT (Sigma-Aldrich, Sweden) at a final concentration of 20 μM and mixed with the desired amount of preformed fibers (0–30 μM). The samples were incubated in 96-well, half-area transparent-bottom plates with a nonbinding surface (CLS3881; Corning, Corning, NY) at 37°C using a plate reader incubator instrument (Fluorostar Optima; BMG Labtech, Ortenberg, Germany). Incubation was in quiescent conditions with fluorescent reading from the bottom of the plate (excitation: 440 nm, emission: 480 nm) at every 5 min. Each experiment included four technical replicates for each condition, and at least three independent experiments were performed.

Seeded aggregation of monomeric aS followed by NMR

Freshly gel-filtered aS monomers in TBS (100 μM) with 5% (*v*/*v*) D_2O were mixed with preformed fiber seeds at desired amounts ($c^{seeds} = 0 \ldots 9.5\ \mu$M) to a final volume of 500 μL. Samples were immediately transferred to the NMR spectrometer, and the successive acquisition of 1D ^{1}H data (real-time NMR) was started after 100 s ($c^{seeds} = 1\ \mu$M) as 70 s ($c^{seeds} = 2\ \mu$M, $c^{seeds} = 5\ \mu$M, $c^{seeds} = 9.5\ \mu$M) at $T = 310$ K after mixing. Data acquisition occurred at an 800 MHz Avance NEO NMR spectrometer (Bruker) operated with a cryogenic QCI probe. Every 1D ^{1}H NMR spectrum was acquired for 277 s. The spectral region of each 1D ^{1}H spectrum was integrated between 0.5 and 2.5 ppm after careful correction of the baseline of corresponding NMR spectra.

Atomic force microscopy

Sonicated preformed fibers were 20 times diluted into Milli-Q water and deposited on freshly cleaved mica. After 10 min, the mica was rinsed with filtered Milli-Q water and dried under a gentle nitrogen stream. Images were recorded on an NTEGRA Prima setup (NT-MDT, Moscow, Russia) using a gold-coated single crystal silicon cantilever (NT-MDT, NSG01, spring constant of ~5.1 N/m) and a resonance frequency of ~180 kHz in tapping mode. 512 × 512-pixel images were acquired with a scan rate of 0.5 Hz. Images were analyzed using the WSxM 5.0 software (30). For characterization of the fiber length, at least nine 10 × 10 μm images were taken in three different areas of the mica. The fibers were automatically identified and measured using flooding analysis of the WSxM software.

Kinetic data analysis

Fitting of experimental kinetic data acquired by ThT fluorescence and NMR spectroscopy was conducted by using Igor Pro 9 (WaveMetrics).

Both ThT and NMR data were normalized regarding the maximum measured signal height in corresponding spectra (for the case of NMR spectroscopy: normalization concerning the start of the aggregation kinetics; for the case of ThT fluorescence spectroscopy: normalization concerning the end of the aggregation kinetics). Single or double exponential functions were used to fit the experimental data to provide kinetic rate constants and amplitudes for the aS elongation reactions. Linear functions were used to determine initial rate constants (v_i) focusing on the first 30 min of the kinetic reactions. Global fitting of kinetic data obtained by ThT fluorescence presented in Fig. S4 was performed using AmyloFit (17) by applying an elongation-only model or an elongation plus secondary nucleation model.

RESULTS AND DISCUSSION

We first used ThT fluorescence to monitor the aggregation process of 100 μM aS monomers in the presence of preformed fibers (seeds) in quiescent conditions at pH 7.4. The amyloid fibers used as seeds were prepared by sonication and found to be on average a few hundred nanometers in length (Fig. S2). At this pH, there is no secondary nucleation taking place during aS amyloid formation (19,20,31), and because of the quiescent conditions used, no primary nucleation nor fiber fragmentation processes occur (Fig. S1 *B*). When the concentration of fiber seeds was varied between 1 μM and 30 μM, the resulting kinetic fluorescence traces (Fig. 1 *A*), reporting on amyloid amount, immediately increase without a lag phase and continue to increase until reaching a stationary phase. Analysis of initial velocities (v_i) of the ThT kinetics at different seed concentrations reveals a linear dependence of v_i on seed concentration (Fig. 1 *C* and *E*) as expected for an elongation process (18). The midpoint (time when reaching 50% of the total increase of ThT fluorescence emission) decreases with increasing concentration of amyloid seeds (Fig. S3), and these parameters display a linear dependence in a double logarithmic plot. The latter linearity demonstrates that the dominant mechanism of the reaction (elongation here) is conserved within the experimental range (1,17). Some aggregated solutions (without ThT) were analyzed for the presence of remaining monomers by measuring 280-nm absorption of samples after removal of formed fibers by centrifugation. Typically, around 10% (10 μM) of the monomers remained at the final stage regardless of seed concentration used.

Next, we used 1D ^{1}H NMR spectroscopy (Fig. S4 *A*) to probe the same amyloid elongation reaction of aS (Fig. 1 *B*), but now following aS monomer disappearance from the soluble state. Whereas soluble aS monomers are directly detected in the NMR spectra, aS amyloid fibrils are invisible due to their large molecular size except for some resonance signals of the polypeptide chain that is still flexible in the fibrils. Like for the ThT signal, the decay of the NMR integral depends on the amyloid seed concentration. Initial rate constants for the NMR data (linear fitting of early time points, Fig. 1 *D*) reveal a similar seed concentration dependence, and magnitudes of v_i, as the ThT-derived data (Fig. 1 *E*). The midpoints of the reactions are similar when detected by ThT fluorescence and by NMR spectroscopy (time when 50% of monomers are gone), and also for NMR, the midpoints decrease with increasing concentration of amyloid seeds (Fig. S3). To assure that the ThT dye did not affect the kinetic reactions probed by fluorescence, we also collected NMR data for an amyloid elongation reaction that included ThT and compared that to the NMR data for an identical aS sample lacking ThT (Fig. S4 *B*). In accord with no effect of ThT, the change in the integral originating from the ThT resonance signal exhibited the same kinetic profile as that originating from the aS monomers.

The above results imply that ThT fluorescence (amyloid formation) and NMR spectroscopic (disappearance of monomers) methods report on the same process, and thus no intermediate states are kinetically resolved (e.g., one could have envisioned monomers disappearing before amyloids appeared). However, global fitting of the kinetic traces from the ThT experiments using an elongation-only model in the web-based fitting program AmyloFit (1,17) did not give a satisfactory fit. Addition of a secondary nucleation component did not improve the fit either (Fig. S5). Similar discrepancies between kinetic data and fits to models are often dismissed due to technical and/or experimental complications (reported, e.g., in (19)).

When considering this "mismatch" further, we uncovered that the ThT fluorescence and the NMR spectroscopic kinetic traces could all be fitted successfully to double exponential functions comprising a slow and a fast phase. In contrast, applying mono-exponential functions did not reliably fit the experimental data (Fig. S6). Important to point out, exponential decay functions have no physical meaning here but are merely a way to probe the number of involved reactions that significantly differ in kinetic rate constants. The amplitude of each phase reports on the amount of monomer consumed before and after the crossover time between fast and slow kinetic phases. The kinetic rate constants and amplitudes for the two kinetic phases match reasonably well comparing NMR spectroscopy with ThT fluorescence at each condition (Fig. 2 *A* and *B*; Table 1).

Since both ThT fluorescence and NMR capture the two kinetic phases and the rate constants as the amplitudes match between the two detection methods, the two kinetic phases must involve monomer disappearance apace with amyloid extension. If the two phases had reported on sequential steps, the NMR kinetics should be faster than the ThT kinetics as monomers must per se disappear before fibers appear. Moreover, the ratios of the amplitudes should not vary with seed concentration if the phases were sequential. Although there is no secondary nucleation expected at these conditions (19,20,31), if it had been associated with the fast phase, it should have been dominant at low seed concentration and not (as here) at high seed concentrations. Also, secondary nucleation requires a lag time to build up sufficient new nuclei whereafter positive curvature is

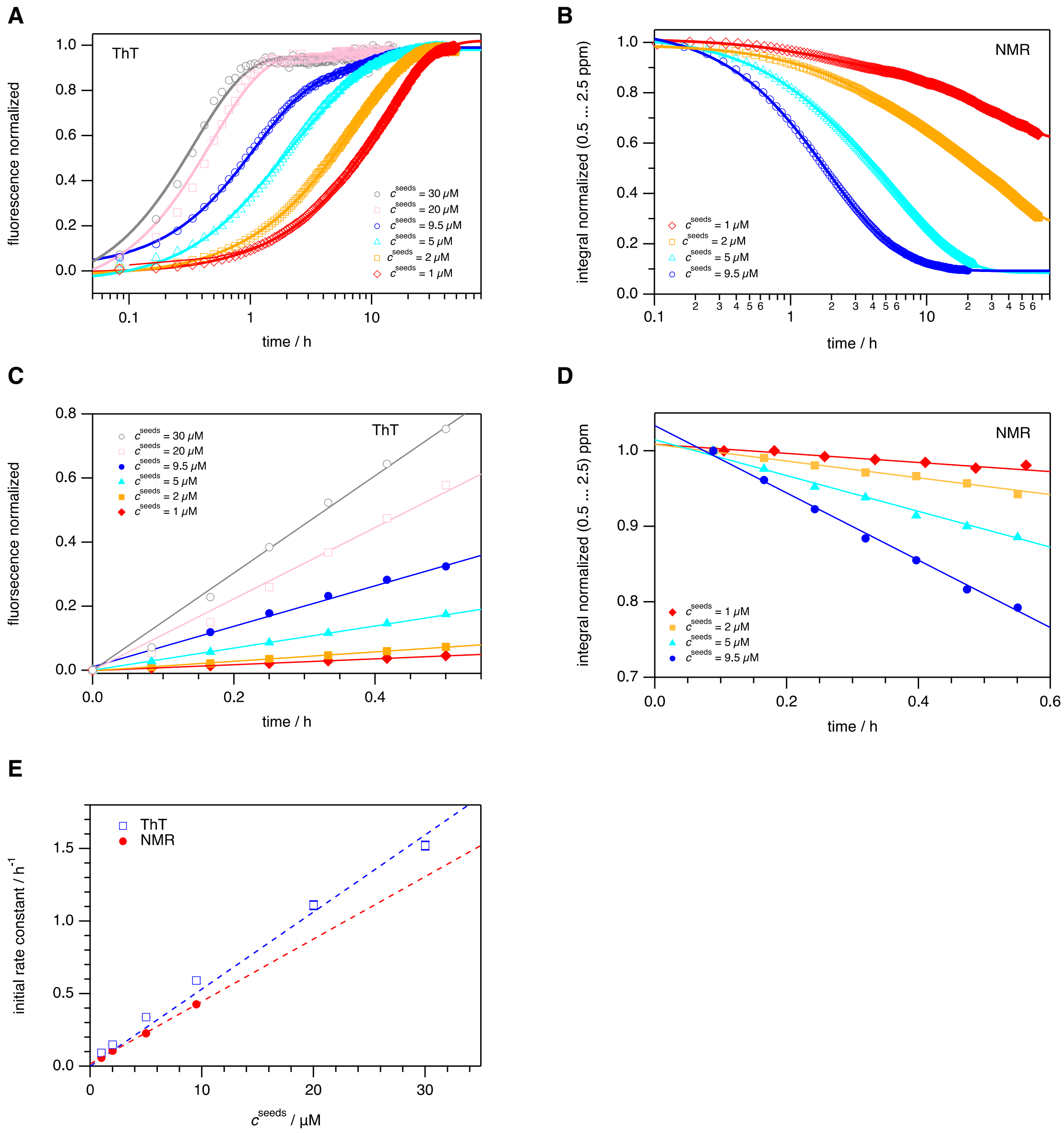

FIGURE 1 Elongation kinetics of aS amyloids monitored by ThT and NMR. Seeded aggregation of 100-µM aS monomers using various concentrations of preformed fibers (seeds) as followed by ThT fluorescence (*A* and *C*) and NMR spectroscopy (*B* and *D*), followed at $T = 310$ K. (*A* and *B*) Concentrations of seeds have been set to the following: $c = 1$ µM (*diamonds, colored in red*), $c = 2$ µM (*rectangles, colored in orange*), $c = 5$ µM (*triangles, colored in cyan*), $c = 9.5$ µM (*circles, colored in blue*), $c = 20$ µM (*squares, colored in pink*), $c = 30$ µM (*circles, colored in gray*). The continuous lines show double exponential fits preserving color coding. Corresponding data are presented in Table 1. The initial rate constants following the aggregation process by ThT fluorescence (*C*) and NMR spectroscopy (*D*) evaluating the first 30 min of the reaction show linear dependence on seed concentration (*E*) and have been determined by linear fitting of the data shown in (*C*) and (*D*), respectively (*open rectangles colored in blue represent ThT fluorescence and solid circles colored in red represent NMR spectroscopic data*).

observed in kinetic traces (19,32,33), but this is not observed here. Explanations for poor global fits have included fiber flocculation and/or fiber precipitation, distorting the fluorescence signal and thereby the kinetics. Such processes are excluded here as the two phases are detected independently by both NMR spectroscopy and ThT fluorescence.

We note that when the results between ThT fluorescence and NMR spectroscopy are numerically compared, the kinetic rate constants (Fig. 1 *E*) are slightly faster, and the midpoint times (Fig. S3) are slightly lower, when determined by ThT fluorescence compared with by NMR spectroscopy. We assign this discrepancy as an artifact due to differences in experimental setups. As monomers must disappear before amyloids appear, ThT kinetics being faster than NMR is inconsistent with any model. Instead, we emphasize the qualitative similarity between the results from two independent methods.

What is the origin of the two kinetic phases? An option is that elongation of the two ends of the fiber seeds display

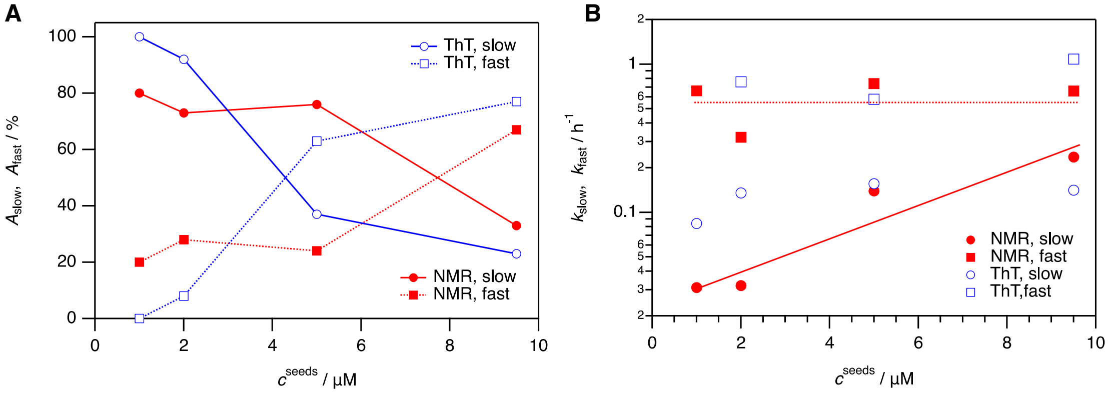

FIGURE 2 Amplitudes and rate constants for fast and slow phases versus seed concentration. Parameters obtained from double exponential fitting of NMR spectroscopic (*colored in red*) as ThT fluorescence data (*colored in blue*) and their dependence on amyloid seed concentration. (*A*) Relative amplitudes of the slow (*circles, open for ThT fluorescence and solid mode for NMR data*) and the fast component (*rectangles, open for ThT fluorescence and solid mode for NMR data*). (*B*) Kinetic rate constants of the slow and the fast component. Color coding and markers as in (*A*). The lines in (*A*) are shown to guide the eye, whereas the lines in (*B*) correspond to fitting of a linear function in a semilogarithmic plot to the data obtained by NMR. Lines in continuous mode highlight the slow phase, and lines in dotted mode highlight the fast phase in (*A*) and (*B*).

different kinetics, which has indeed been suggested in some studies (34). However, this scenario is excluded as then the amplitudes should not vary with seed concentration (the ratio of fibril ends will be 50:50 at each seed concentration). Another attractive option is the presence of two parallel paths: a slow, direct addition of monomers to fiber ends and a fast, surface-mediated channeling of monomers to fiber ends. Indeed, transient interactions between aS monomers and amyloid fiber surfaces have been reported (32) at conditions that did not allow for elongation. Also, for another amyloidogenic protein, β2-microglobulin, fiber-surface binding of monomers was proposed as an intermediate in a sequential elongation mechanism (35), and there is quartz crystal microbalance and surface plasmon resonance data for amyloid elongation that cannot be explained without rapid binding of monomers to fiber surfaces (24,36,37). In addition to in vitro experimental support, also recent in silico multiscale simulations of amyloid-β_{16-22} aggregation unraveled short-lived monomer-fiber surface interactions (termed "nonregistered sites") that contributed to successful elongation (38). However, based on mathematical modeling (Supporting material), we exclude this mechanism as the reason for the bi-phasic kinetic behavior observed here. The dependence on monomer concentration in a two pathways model will not display as two exponential functions. We also excluded the stop-and-go type of behavior

TABLE 1 Kinetic parameters for experimental ThT and NMR elongation data

NMR	$c^{seed} = 1\ \mu M$	$c^{seed} = 2\ \mu M$	$c^{seed} = 5\ \mu M$	$c^{seed} = 9.5\ \mu M$
y_0	0.599 ± 0.001	0.250 ± 0.001	0.086 ± 0.001	0.093 ± 0.001
A_{slow}	0.333 ± 0.001	0.542 ± 0.001	0.720 ± 0.001	0.32 ± 0.01
A_{fast}	0.084 ± 0.001	0.201 ± 0.001	0.223 ± 0.001	0.65 ± 0.01
k_{slow}/h^{-1}	0.031 ± 0.001	0.032 ± 0.001	0.139 ± 0.001	0.236 ± 0.005
k_{fast}/h^{-1}	0.66 ± 0.02	0.321 ± 0.005	0.739 ± 0.005	0.659 ± 0.008
$t_{midpoint}$/h[a]	13	9.3	3.0	1.1
**ThT**				
y_0	1.019 ± 0.002	0.992 ± 0.001	0.987 ± 0.001	0.987 ± 0.001
A_{slow}	−1.01 ± 0.01	−0.919 ± 0.006	−0.367 ± 0.009	−0.231 ± 0.003
A_{fast}	*n.d.*	−0.085 ± 0.006	−0.631 ± 0.008	−0.764 ± 0.003
k_{slow}/h^{-1}	0.084 ± 0.001	0.135 ± 0.001	0.156 ± 0.002	0.141 ± 0.002
k_{fast}/h^{-1}	*n.d.*	0.76 ± 0.09	0.579 ± 0.008	1.08 ± 0.01
$t_{midpoint}$/h	8.3	5	2	1

Results of fitting a double exponential function $y(t) = y_0 + A_{slow}\exp(-k_{slow}\ t) + A_{fast}\exp(-k_{fast}\ t)$ to the experimental data determined by ThT fluorescence and NMR spectroscopy of samples with 100 μM aS monomers and varying concentration of preformed fibers (seeds), at $T = 310$ K. The midpoints, $t_{midoint}$, are plotted in Fig. S3. In Fig. 2, the amplitudes of the two phases are reported as percent of total amplitude. Amplitudes and rate constants for the fast and the low phases are plotted as a function of added seed concentration in Fig. 2. n.d.: not detected.

[a]To determine midpoints of NMR kinetics, the time dependence of integrals obtained in 1D ^{1}H NMR spectra (Fig. 1 B) were converted into the concentration of aS monomers. Thus, $t_{midpoint}$ has been obtained at $c^{aS} = 50\ \mu$M. To determine the final amount of aS monomers after seeded elongation reactions, samples were spun down, and monomers left in solution were determined by absorption spectroscopy.

as another potential explanation for the kinetic data (Supporting material).

Instead, we analyzed a third possibility, the disordered aggregation model, that has been proposed (28) but not demonstrated experimentally before. For this, we inspected the kinetic ThT traces (for which we probed the largest range in seed concentration) in more detail. When we plot the fibril conversion rate per seed as a function of the monomer concentration (Fig. 3 *A*; Supporting material), we see excellent data collapse, apart from the noisy behavior at long times in the experiments with low seed concentrations. This collapse supports the notion that the transition from fast to slow kinetics is a function of monomer concentration, not time (as is expected for the stop-and-go model). Fig. 3 *A* reveals two distinct linear regimes with different monomer concentration dependence. Above monomer concentrations of 20 μM, the fiber elongation rate scales linearly with concentration. Below this concentration, the elongation rate depends less, or not at all, on monomer concentrations.

Previous experiments (19,40–44) and theory (28,40) have shown two-phase elongation kinetics with a linear, diffusion-limited regime at low concentration followed by a flat plateau when the conformational rearrangement becomes limiting for the kinetics (no dependence on monomer concentration) (Fig. 3 *B*). The diffusion-limited regime occurs when the arrival of molecules is slow compared with the conformational search. Mathematical calculations for the molecular system probed here (Supporting material) demonstrate that diffusive arrival is always faster than 10^4 s^{-1}, whereas elongations are always slower than 1 s^{-1}. We therefore propose that the slow growth regime observed in our experiments is consistent with conformationally limited plateau kinetics. The fast growth regime at higher monomer concentrations is explained by a molecule deposition rate that overwhelms the conformational search, resulting in disordered monomers incorporated at the fiber end (28,39,45) (Fig. 3 *B*).

We note that typical initial-rate analysis of elongation as a function of monomer concentration often reveals apparent saturation kinetics at high monomer concentrations. However, such analysis involves linear fitting of a set of data points that cover a certain time span, and in addition, early kinetic data points are missed due to mixing time, pipetting into plate reader, instrument dead time, etc. The faster the elongation reaction is, the more of the early reaction is not captured in the analysis; thereby, the disordered regime is easily missed.

CONCLUSIONS

The collected data by NMR and fluorescence spectroscopy, probing different aspects of the amyloid fiber growth reaction, reveal that elongation of aS amyloids is governed by two apparent kinetic phases in vitro at physiological pH. Careful analysis and testing of various possible mechanisms allowed us to reveal the underlying molecular mechanism for this experimental result. We discovered the presence of two kinetic regimes that depend differentially on instantaneous monomer concentration. At low monomer concentrations, elongation appears conformational-change limited; at higher monomer concentrations, rapid disordered growth occurs (Fig. 3 *B*). In the case of amyloid fibers, the conformational search is limited by the search over β-sheet alignments (38,46), so disorder will involve misaligned β-strands (28,39). Although the amyloid

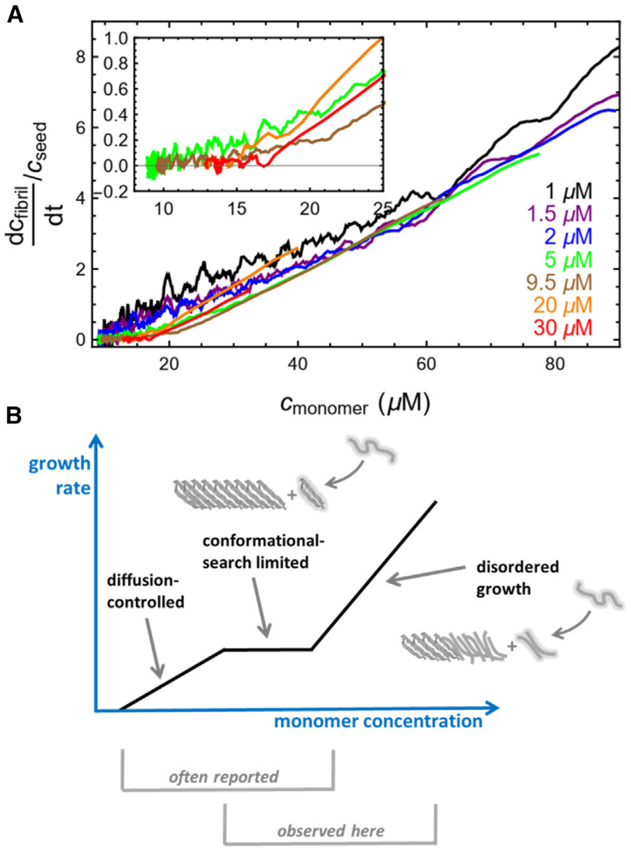

FIGURE 3 Amyloid growth rate as a function of monomer concentration. (*A*) Plot of amyloid fiber elongation rate per seed as a function of monomer concentration obtained from the ThT kinetic data (units: hr^{-1}). The curves have been smoothed by averaging over a window of ±4 adjacent data points. Inset shows a zoom-in on the most reliable data (removing the noise-dominated behaviors of 1- and 2-μM seeds). The data collapse shows distinct kinetic behaviors above and below 20 μM monomers. (*B*) Schematic illustration showing kinetic regimes of amyloid elongation. At low monomer concentrations, growth is diffusion controlled, and at higher monomer concentration, growth becomes limited by the conformational search to find the right β-strand alignment. This regime shows weak dependence on monomer concentration. Eventually, at higher monomer concentrations, growth rates increase with monomer concentration as elongation becomes disordered, meaning that monomers are deposited faster than the conformational search can be completed (28,39). This "disorder" may only involve a few residues because the molecules are most likely to become trapped in states that resemble the ordered state. In previous studies, the two first regimes have been reported, whereas in this study, we show that aS elongation in these conditions involves the last two regimes.

core of α-synuclein, roughly 50 residues, together with the remaining 90 termini residues can misalign in many hundred different ways, the slope of the fast regime shown in Fig. 3 *A* is only about fivefold greater than the slope of the slow regime. Therefore, the misalignments are likely limited to just a few amino acids. Possibly the disorder relates to increased fraying of the ordered amyloid core at the N- and C-terminal ends by a few residues. This observed behavior may be one factor among others that govern observed variations in amyloid fold and stability. Studies are underway to structurally explore α-synuclein amyloids formed in the proposed "disordered" elongation regime.

SUPPORTING MATERIAL

Supporting material can be found online at https://doi.org/10.1016/j.bpj.2023.05.009.

AUTHOR CONTRIBUTIONS

I.H., M.K., and P.W.S. designed the study; I.H., H.W., and M.K. performed experiments; J.D.S. performed mathematical modeling; J.D.S., I.H., M.K., and P.W.S. analyzed data; J.D.S., I.H., M.K., and P.W.S. wrote the paper.

ACKNOWLEDGMENTS

We thank Fabian Immler for supporting the acquisition of NMR spectroscopic data and Ranjeet Kumar for protein production. The Knut and Alice Wallenberg Foundation and the Swedish Research Council are acknowledged for funding (P.W.S.). J.D.S. acknowledges support from NIH grant R01GM141235. We also thank the University of Konstanz for the permanent investment into the NMR infrastructure.

DECLARATION OF INTERESTS

The authors declare no competing interests.

REFERENCES

1. Chiti, F., and C. M. Dobson. 2017. Protein misfolding, amyloid formation, and human disease: a summary of progress over the last decade. *In* Annual Review of Biochemistry, *Vol 86*. R. D. Kornberg, ed. Annual Reviews, pp. 27–68.
2. Otzen, D., and R. Riek. 2019. Functional amyloids. *Cold Spring Harb. Perspect. Biol.* 11, a033860.
3. Iadanza, M. G., M. P. Jackson, …, S. E. Radford. 2018. A new era for understanding amyloid structures and disease. *Nat. Rev. Mol. Cell Biol.* 19:755–773.
4. Jarrett, J. T., E. P. Berger, and P. T. Lansbury. 1993. The carboxy terminus of the beta-amyloid protein IS critical for the seeding of amyloid formation - implications for the pathogenesIS of alzheimers-dISease. *Biochemistry.* 32:4693–4697.
5. Wakabayashi, K., K. Matsumoto, …, H. Takahashi. 1997. NACP, a presynaptic protein, immunoreactivity in Lewy bodies in Parkinson's disease. *Neurosci. Lett.* 239:45–48.
6. Cooper, G. J., A. C. Willis, …, K. B. Reid. 1987. Purification and characterization of a peptide from amyloid-rich pancreases of TYPE-2 diabetic-patients. *Proc. Natl. Acad. Sci. USA.* 84:8628–8632.
7. John, A., and W. van der Pluijm. 2018. The global prevalence of Parkinson's disease over the next ten years. *Ann. Neurol.* 84:S219.
8. Elkouzi, A., V. Vedam-Mai, …, M. S. Okun. 2019. Emerging therapies in Parkinson disease - repurposed drugs and new approaches. *Nat. Rev. Neurol.* 15:204–223.
9. Goldberg, M. S., and P. T. Lansbury. 2000. Is there a cause-and-effect relationship between alpha-synuclein fibrillization and Parkinson's disease? *Nat. Cell Biol.* 2:E115–E119.
10. Spillantini, M. G., M. L. Schmidt, …, M. Goedert. 1997. α-Synuclein in Lewy bodies. *Nature.* 388:839–840.
11. Uversky, V. N. 2007. Neuropathology, biochemistry, and biophysics of α-synuclein aggregation. *J. Neurochem.* 103:17–37.
12. Polymeropoulos, M. H., C. Lavedan, …, R. L. Nussbaum. 1997. Mutation in the α-synuclein gene identified in families with Parkinson's disease. *Science.* 276:2045–2047.
13. Pfefferkorn, C. M., Z. Jiang, and J. C. Lee. 2012. Biophysics of α-synuclein membrane interactions. *Biochim. Biophys. Acta.* 1818:162–171.
14. Fusco, G., T. Pape, …, A. De Simone. 2016. Structural basis of synaptic vesicle assembly promoted by α-synuclein. *Nat. Commun.* 7, 12563.
15. Dev, K. K., K. Hofele, …, H. van der Putten. 2003. Part II: α-synuclein and its molecular pathophysiological role in neurodegenerative disease. *Neuropharmacology.* 45:14–44.
16. Lassen, L. B., L. Reimer, …, P. H. Jensen. 2016. Protein partners of α-synuclein in health and disease. *Brain Pathol.* 26:389–397.
17. Meisl, G., J. B. Kirkegaard, …, T. P. J. Knowles. 2016. Molecular mechanisms of protein aggregation from global fitting of kinetic models. *Nat. Protoc.* 11:252–272.
18. Buell, A. K. 2019. The growth of amyloid fibrils: rates and mechanisms. *Biochem. J.* 476:2677–2703.
19. Buell, A. K., C. Galvagnion, …, C. M. Dobson. 2014. Solution conditions determine the relative importance of nucleation and growth processes in alpha-synuclein aggregation. *Proc. Natl. Acad. Sci. USA.* 111:7671–7676.
20. Gaspar, R., G. Meisl, …, S. Linse. 2017. Secondary nucleation of monomers on fibril surface dominates α-synuclein aggregation and provides autocatalytic amyloid amplification. *Q. Rev. Biophys.* 50:e6.
21. Guo, J. L., and V. M. Y. Lee. 2014. Cell-to-cell transmission of pathogenic proteins in neurodegenerative diseases. *Nat. Med.* 20:130–138.
22. Gade Malmos, K., L. M. Blancas-Mejia, …, D. Otzen. 2017. ThT 101: a primer on the use of thioflavin T to investigate amyloid formation. *Amyloid.* 24:1–16.
23. Buell, A. K., A. Dhulesia, …, M. E. Welland. 2012. Detailed analysis of the energy barriers for amyloid fibril growth. *Angew. Chem. Int. Ed. Engl.* 51:5247–5251.
24. Cannon, M. J., A. D. Williams, …, D. G. Myszka. 2004. Kinetic analysis of beta-amyloid fibril elongation. *Anal. Biochem.* 328:67–75.
25. Wördehoff, M. M., O. Bannach, …, E. Birkmann. 2015. Single fibril growth kinetics of α-synuclein. *J. Mol. Biol.* 427:1428–1435, Part B.
26. Pinotsi, D., A. K. Buell, …, C. F. Kaminski. 2014. Direct observation of heterogeneous amyloid fibril growth kinetics via two-color super-resolution microscopy. *Nano Lett.* 14:339–345.
27. Ferkinghoff-Borg, J., J. Fonslet, …, M. H. Jensen. 2010. Stop-and-go kinetics in amyloid fibrillation. *Phys. Rev.* 82, 010901.
28. Schmit, J. D. 2013. Kinetic theory of amyloid fibril templating. *J. Chem. Phys.* 138, 185102.
29. Werner, T., R. Kumar, …, P. Wittung-Stafshede. 2018. Abundant fish protein inhibits α-synuclein amyloid formation. *Sci. Rep.* 8
30. Horcas, I., R. Fernández, …, A. M. Baro. 2007. WSXM: a software for scanning probe microscopy and a tool for nanotechnology. *Rev. Sci. Instrum.* 78, 013705.
31. Horvath, I., R. Kumar, and P. Wittung-Stafshede. 2021. Macromolecular crowding modulates α-synuclein amyloid fiber growth. *Biophys. J.* 120:3374–3381.

32. Kumari, P., D. Ghosh, …, C. Eichmann. 2021. Structural insights into α-synuclein monomer-fibril interactions. *Proc. Natl. Acad. Sci. USA*. 118.

33. Lorenzen, N., S. I. A. Cohen, …, D. Otzen. 2012. Role of elongation and secondary pathways in S6 amyloid fibril growth. *Biophys. J.* 102:2167–2175.

34. Heldt, C. L., S. Zhang, and G. Belfort. 2011. Asymmetric amyloid fibril elongation: a new perspective on a symmetric world. *Proteins*. 79:92–98.

35. Chatani, E., R. Ohnishi, …, Y. Goto. 2010. Pre-steady-state kinetic analysis of the elongation of amyloid fibrils of β2-microglobulin with tryptophan mutagenesis. *J. Mol. Biol.* 400:1057–1066.

36. Esler, W. P., E. R. Stimson, …, J. E. Maggio. 2000. Alzheimer's disease amyloid propagation by a template-dependent dock-lock mechanism. *Biochemistry*. 39:6288–6295.

37. Ogi, H., Y. Fukunishi, …, M. Hirao. 2011. Seed-dependent deposition behavior of Aβ peptides studied with wireless quartz-crystal-microbalance biosensor. *Anal. Chem.* 83:4982–4988.

38. Jia, Z., J. D. Schmit, and J. Chen. 2020. Amyloid assembly is dominated by misregistered kinetic traps on an unbiased energy landscape. *Proc. Natl. Acad. Sci. USA*. 117:10322–10328.

39. Huang, C., E. Ghanati, and J. D. Schmit. 2018. Theory of sequence effects in amyloid aggregation. *J. Phys. Chem. B*. 122:5567–5578.

40. Buell, A. K., J. R. Blundell, …, T. P. J. Knowles. 2010. Frequency factors in a landscape model of filamentous protein aggregation. *Phys. Rev. Lett.* 104, 228101.

41. Knowles, T. P. J., W. Shu, …, M. E. Welland. 2007. Kinetics and thermodynamics of amyloid formation from direct measurements of fluctuations in fibril mass. *Proc. Natl. Acad. Sci. USA*. 104:10016–10021.

42. van der Wateren, I. M., T. P. J. Knowles, …, C. Galvagnion. 2018. C-terminal truncation of alpha-synuclein promotes amyloid fibril amplification at physiological pH. *Chem. Sci.* 9:5506–5516.

43. Collins, S. R., A. Douglass, …, J. S. Weissman. 2004. Mechanism of prion propagation: amyloid growth occurs by monomer addition. *PLoS Biol.* 2:e321.

44. Lomakin, A., D. S. Chung, …, D. B. Teplow. 1996. On the nucleation and growth of amyloid beta-protein fibrils: detection of nuclei and quantitation of rate constants. *Proc. Natl. Acad. Sci. USA*. 93:1125–1129.

45. Whitelam, S., Y. R. Dahal, and J. D. Schmit. 2016. Minimal physical requirements for crystal growth self-poisoning. *J. Chem. Phys.* 144, 064903.

46. Jia, Z., A. Beugelsdijk, …, J. D. Schmit. 2017. The levinthal problem in amyloid aggregation: sampling of a flat reaction space. *J. Phys. Chem. B*. 121:1576–1586.

Biophysical Journal, Volume *122*

Supplemental information

Distinct growth regimes of α-synuclein amyloid elongation

Istvan Horvath, Hannah Welte, Jeremy D. Schmit, Michael Kovermann, and Pernilla Wittung-Stafshede

Supplemental Information

Distinct growth regimes of α-synuclein amyloid elongation

Istvan Horvath, Hannah Welte, Jeremy D. Schmit, Michael Kovermann, and
Pernilla Wittung-Stafshede

Content:

Figures S1-S6 pages 2-7

Theory pages 8-11

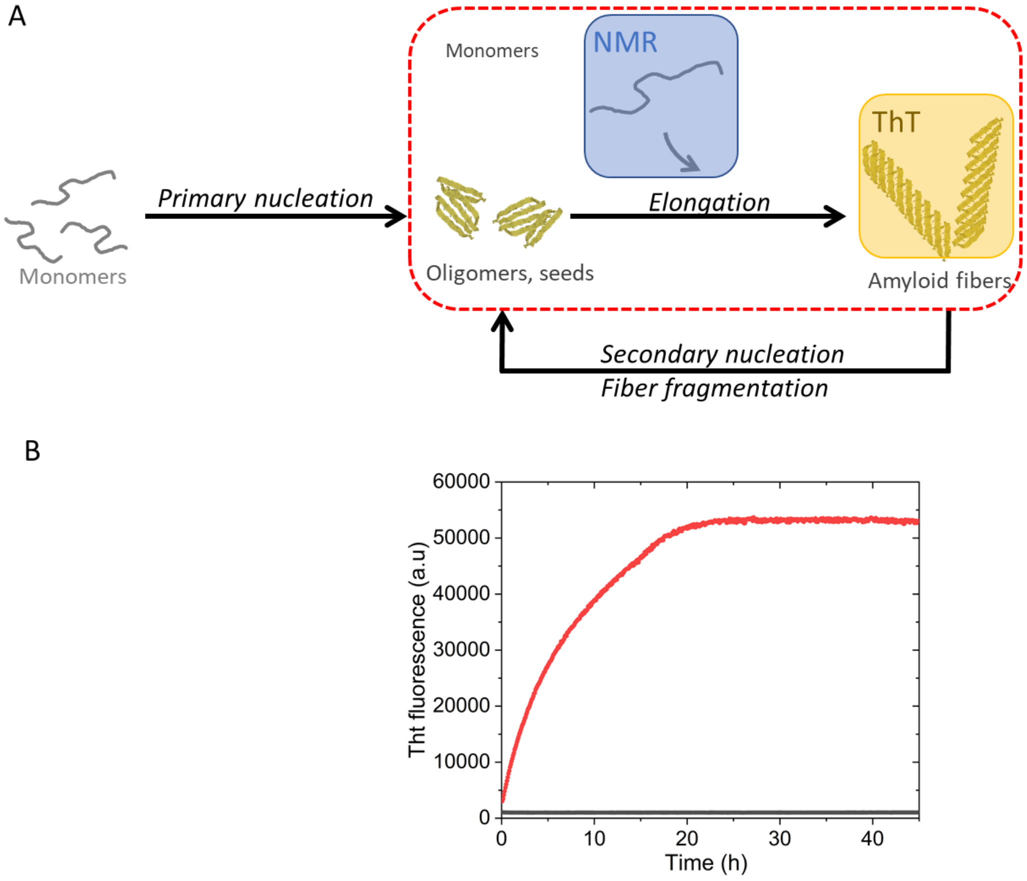

Figure S1

(A) Amyloid formation mechanism including primary and secondary processes highlighting the methods (NMR, ThT) and pathways (red box; elongation) used in this study. (B) Incubation of 100 µM monomers in the absence (black) and in the presence of 1 µM pre-formed amyloid fiber seeds (red) in quiescent conditions, showing lack of primary nucleation within the time window measured in the absence of seeds.

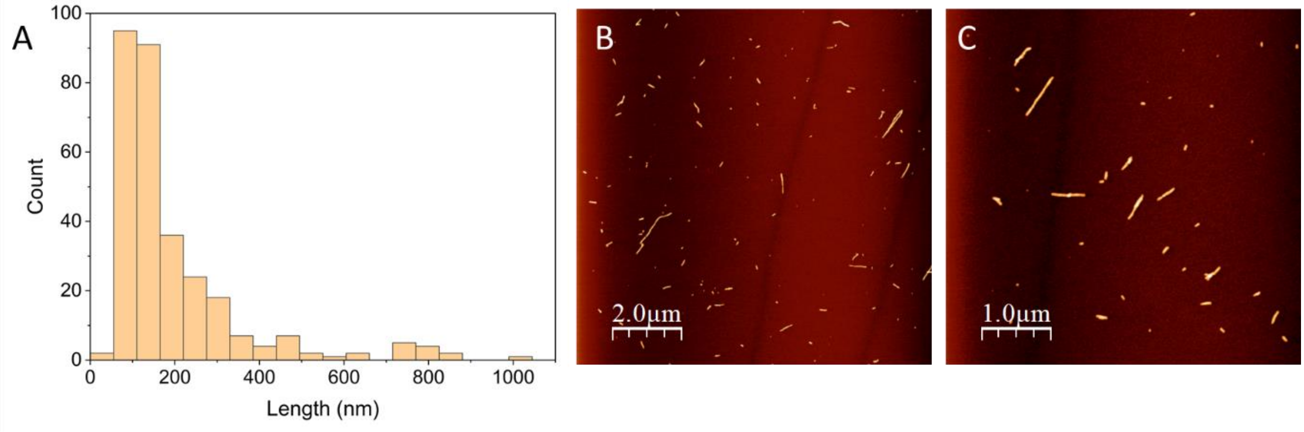

Figure S2

Size characterization of the used amyloid fiber seeds by AFM. Histogram of fiber lengths as determined from AFM images (mean length: 195 nm ±162 nm, median: 139 nm) (A), and two representative AFM images of the seeds, Z-range is set to 0-10 nm (B, C).

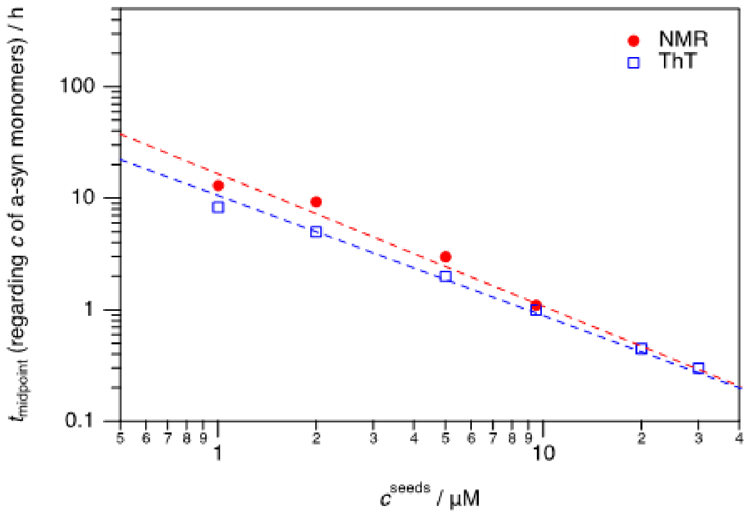

Figure S3

Double logarithmic plot of the midpoints of the seeded aggregation kinetics of aS monomers, $t_{midpoint}$, versus the concentration of pre-formed fiber amyloid seeds, c^{seeds}. The midpoints were determined from NMR spectroscopy (circles, red) and ThT fluorescence (rectangles, blue) experiments using 100 μM aS monomers, at T = 310 K (see **Table 1**). The dashed lines are linear fits (in the double logarithmic plot) to the data sets.

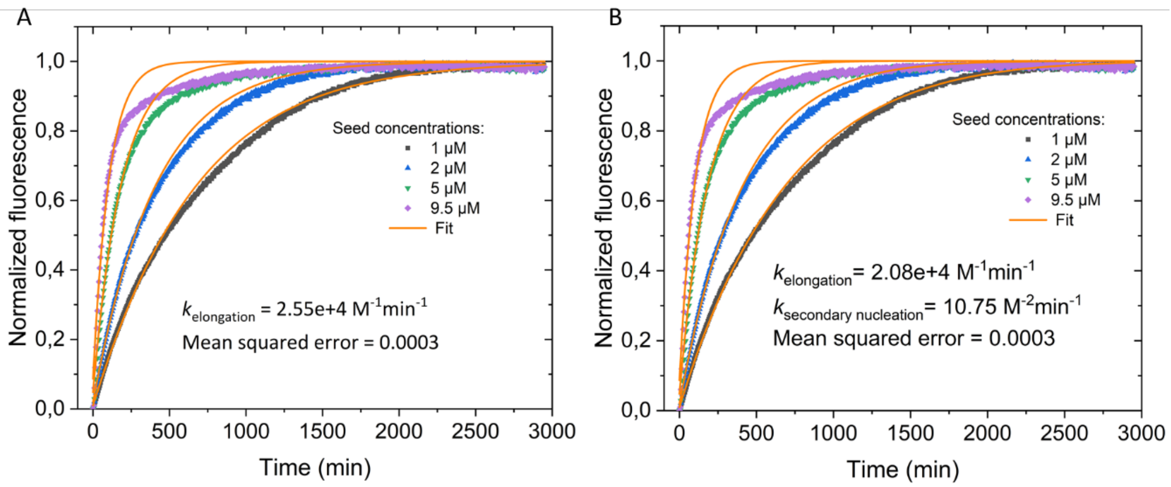

Figure S4

Global analysis by Amylofit of seeded aggregation of 100 μM aS monomers probed by ThT fluorescence at varying amyloid seed concentration. Two different fitting approaches were pursued here: elongation only (A) and elongation combined with secondary nucleation (B). Fitted curves are highlighted in orange in (A) and (B). For each condition, four replicates of experimental data were used for fitting; the plotted experimental curves are the averages.

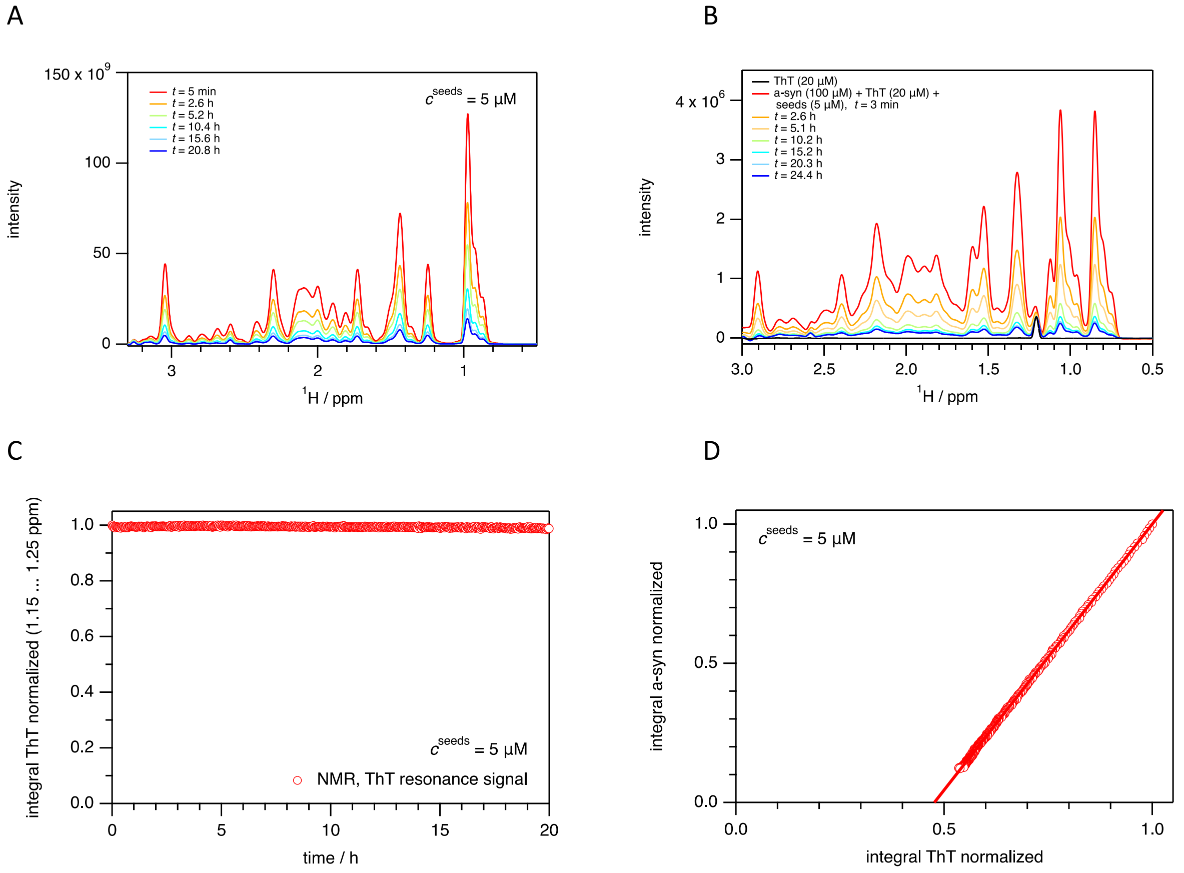

Figure S5

NMR spectroscopic data for aS and ThT at different conditions, at T = 310 K. (A) Real time 1D ^{1}H NMR data following the aggregation process of aS monomers at a concentration of 100 μM in presence of 5 μM amyloid seeds, in absence of ThT. The color coding refers to distinct time points within the series of acquired spectra. Corresponding data are shown in **Figure 1B**. (B) Real time 1D ^{1}H NMR data following the aggregation process of aS monomers at a concentration of 100 μM in presence of 5 μM seeds and in presence of 20 μM ThT. The spectrum colored in black represents the chemical shift observed for ThT. Note that aS has been ^{13}C isotopically enriched here enabling the separate analysis of the resonance signal of ThT within the series of acquired 1D ^{1}H NMR spectra. (C) The integral originating from the resonance signal of ThT was also followed in real time for a sample composed of 20 μM ThT and 5 μM seeds only (control; no change in integral reporting on ThT). (D) NMR data analysis of the aggregation process of aS monomers was conducted by determining the integral of resonance signals arising from the aS only sample (y-axis) and the sample comprising aS and ThT (x-axis). Both experimental set ups give the same numerical parameters for A_{slow}, A_{fast}, k_{slow} and k_{fast}. The continuous line colored in red represents this correlation leading to the conclusion that ThT does not affect aS elongation kinetics.

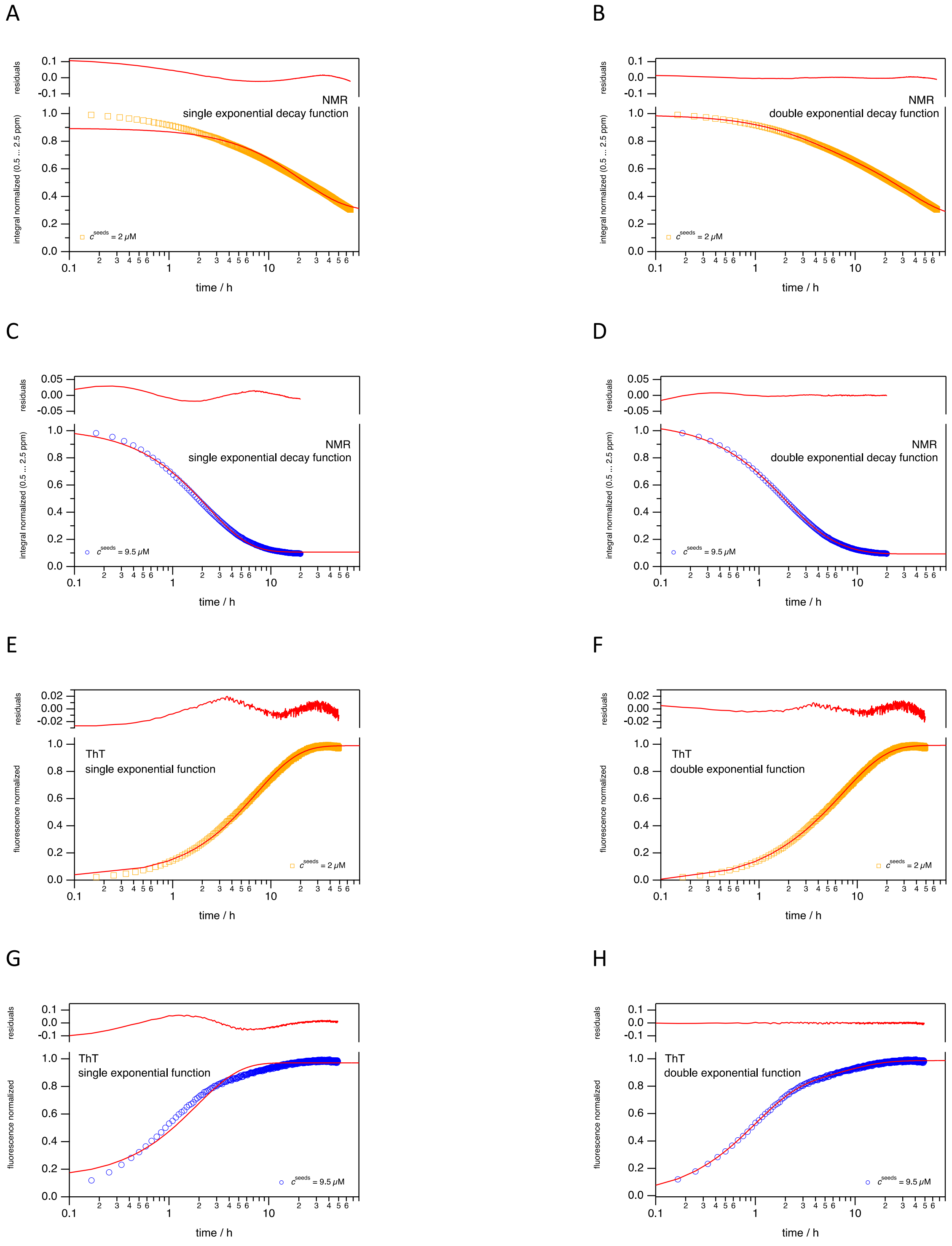

Figure S6

Fitting of the experimental data with exponential functions comprising one (A, C, E, G) or two kinetic phases (B, D, F, H). Fitting results are shown for data obtained by ThT fluorescence (E- H) and by NMR spectroscopy (A- D). The seeded aggregation of aS monomers was followed in presence of pre-formed fibers at a concentration of 2 μM (A, B, E, F) as of 9.5 μM (C, D, G, H). The progression of residuals depends significantly on the number of kinetic phases used in the fitting procedures. So bi-phasic fits giving significant improved match to experimental data compared to mono-phasic fits.

Theory

We considered three amyloid fiber elongation models to explain the biexponential behavior that we observed in our experiments. Our mathematical modelling is explained below:

1. Two pathways model

One possibility is that the two exponential regimes are indicative of different pathways by which monomers can attach to the end of a fibril. Simulations (1) have shown that monomer deposition can occur either by direct attachment from the solution or by first adhering to the side of the fibril and diffusing laterally to the end. The increase in fibril mass comes from the sum of the two pathways

$$\frac{dc_{fibril}}{dt} = J_{direct} + J_{side}$$

Where

$$J_{direct} = k_{direct} c_{sol} c_{end}$$
$$J_{side} = k_{side} \rho_{side} c_{end}$$

The direct pathway is proportional to the concentration of fibril ends c_{end}, the concentration of soluble protein c_{sol}, and a rate constant k_{direct}. Similarly, the side pathway will be proportional the concentration of protein bound to the sides of the fibril. However, because the concentration of binding sites per length of fibril is unknown, we have expressed the side concentration as a dimensionless density, ρ_{side}. This means that the rate constant for side pathway growth, k_{side}, has different units than k_{direct}.

It is an excellent approximation to treat c_{sol} and ρ_{side} in the pseudo-equilibrium limit. To see this, we can compare the molecular collision rate to the growth rate of the fibril. A random coil polypeptide chain has a radius of ~2.5 nm (2), which gives a Stokes-Einstein diffusion constant of $D = 10^2\ \mu m^2/s$. Inserting this value in the Smoluchowski formula gives $(4\pi D a c_{sol})^{-1} \cong 10^5/s$ as the collision rate, where we have a used a target radius of $a = 2$nm and the maximum concentration of $c_{sol} = 100\ \mu M$. To estimate the fibril elongation rate, we need to know the concentration of fibril ends. AFM measurements give an average length of 195 nm, which works out to about 830 molecules per seed using 0.47 nm per layer and 2 molecules per layer. Accounting for the fact that there are two growing ends per fibril gives $c_{end} = c_{seed}/415$. To estimate the initial elongation rate, we fit the first ten ThT measurements to the function $c_{sol} = (100\ \mu M) Exp[-k_{app} t]$ to find the apparent decay rate (**Theory Figure 1 top**).

The fitted rate constants show a linear trend with seed concentration $\frac{k_{app}}{c_{seed}} = 0.081/\mu M/hr$ (**Theory Figure 1 bottom**).

The initial fibril conversion rate is $\frac{dc_{fibril}}{dt} = (100\ \mu M) k_{app}$, which gives a conversion rate per end $\frac{(100\ \mu M) k_{app}}{c_{end}} = 0.93/s$. Because this is 5 orders of magnitude slower than the collision rate, the monomer distribution along the fiber surface will re-equilibrate between monomer addition events to the fiber ends. Therefore, no difference in monomer concentration along the fibers needs to be considered.

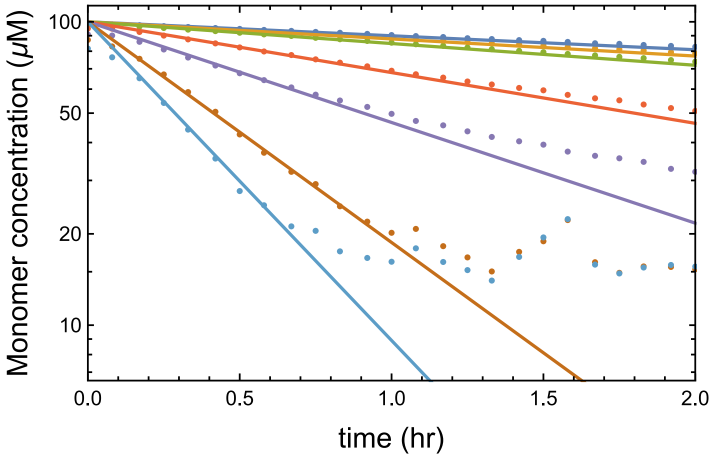

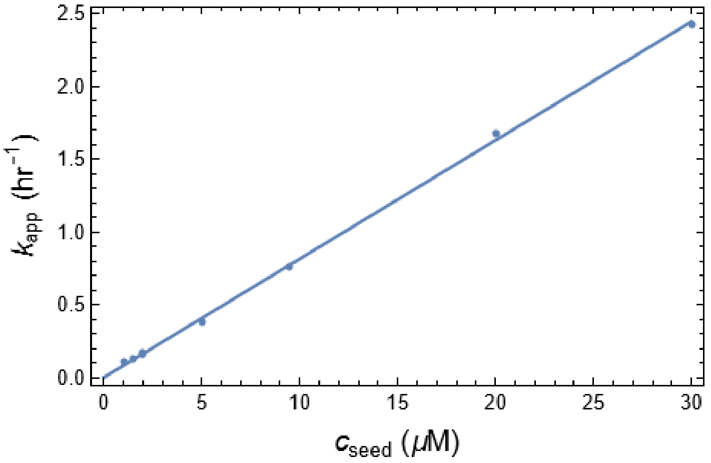

Theory Figure 1. ***Top:*** Fits of the initial fibril elongation rate to $c_{sol} = (100\ \mu M)Exp[-k_{app}t]$. ***Bottom:*** The apparent rate constants obtained from this fitting scale linearly with the seed concentration.

To find an expression for ρ_{side} we assume that there is a concentration c_{site} of binding sites on the side of fibrils, of which c_{bound} are bound to a monomer. The dissociation constant for the side binding sites is given by $k_d = c_{sol}(c_{site} - c_{bound})/c_{site}$, which can be rearranged to give $\rho_{side} = c_{bound}/c_{site} = c_{sol}/(k_d + c_{sol})$.
Inserting this into Equations [1-3], we have

$$\frac{dc_{fibril}}{dt} = c_{end}(k_{direct}c_{sol} + k_{side}\frac{c_{sol}}{k_d + c_{sol}})$$

It is instructive to look at two limits of this equation. If $c_{sol} \ll k_d$ we have

$$\frac{dc_{fibril}}{dt} \cong c_{end}(k_{direct}c_{sol} + k_{side}\frac{c_{sol}}{k_d})$$
$$\frac{dc_{fibril}}{dt} \cong c_{sol}c_{end}(k_{direct} + \frac{k_{side}}{k_d})$$
$$\frac{dc_{fibril}}{dt} \cong c_{sol}k_{app}$$

Where $k_{app} = c_{end}(k_{direct} + \frac{k_{side}}{k_d})$. Thus, at low concentration the two pathways merge to give a single effective conversion rate. In the opposite limit we have $c_{sol} \gg k_d$, which gives

$$\frac{dc_{fibril}}{dt} \cong c_{end}(k_{direct}c_{sol} + k_{side})$$

To get a "fast" elongation regime that is distinct from the slow elongation at low concentration it is necessary that $k_{side} \gg k_{direct}c_{sol}$. However, in this limit the fibril elongation rate is independent of the monomer concentration, which results in linear, not exponential, kinetics. Thus, this model does not support the observed biexponential kinetics.

2. Stop-and-go model

Another possible explanation for biexponential behavior is "stop and go" growth kinetics in which fibril ends switch between growing and arrested states. This could explain biexponential kinetics if all fibrils started in the growing state and gradually converge to a steady state population of arrested ends. This model predicts that the transition to the slow phase is time dependent (all systems will start fast and transition to the slow phase with a time dependence that is independent of the seed concentration), which contrasts to the "two pathways" model above which predicts a transition that depends on the monomer concentration. To evaluate this, we plot the fibril conversion rate per seed as a function of the monomer concentration (**Figure 3**, main manuscript; again, shown below as **Theory Figure 2**). Apart from the noisy behavior at long times in the low seed experiments, we see excellent data collapse, which supports the notion that the transition from fast to slow kinetics is a function of concentration, not time. The common break point between slow and fast regimes (around 20 μM) in **Theory Figure 2** corresponds to very different reaction times between the experiments shown (the midpoints of the seeded reactions span more than one order of magnitude (see **Figure 1A**, **Table 1** in main manuscript).

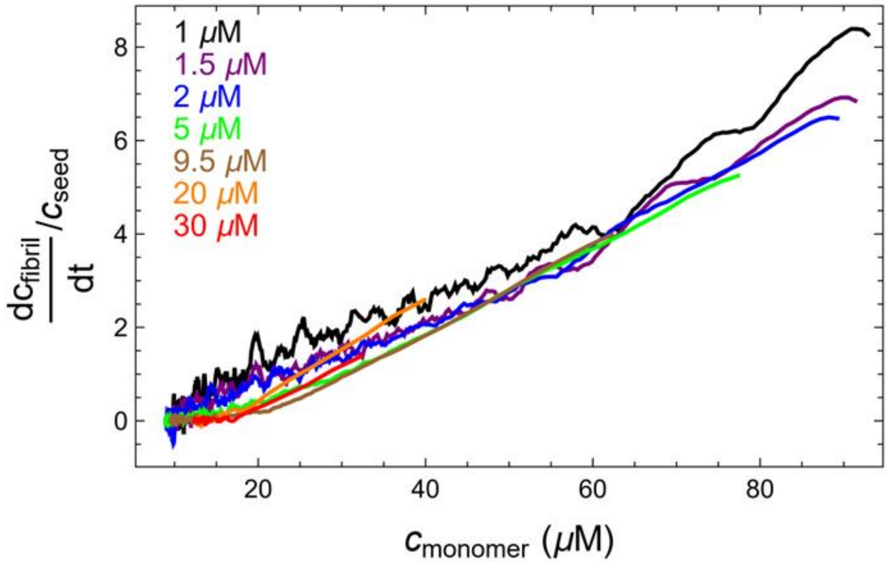

Theory Figure 2. Plot of fibril conversion rate per seed as a function of monomer concentration obtained from the ThT kinetic data (units: hr^{-1}). The curves have been smoothed by averaging over a window of ± 4 adjacent data points.

3. Disordered aggregation model.

With two pathways and stop-and-go disfavored, we turned to a third possible model, termed 'disordered aggregation model'. In **Theory Figure 2**, we see that the fibril elongation rate scales linearly with concentration when the monomer concentration is greater than about 20 μM. The conversion rates slow considerably at concentrations less than this and ultimately reach zero at the saturation monomer concentration near 10 μM. Two kinetic regimes with different monomer-dependence agree with the observed experimental biphasic behavior. We propose (see also main text discussion) that the slow growth regime observed in our experiments is consistent with the plateau

regime; the conformational search is too slow for the diffusion-limited regime to appear under these conditions. The fast growth regime would then be explained by a molecule deposition rate that overwhelms the conformational search resulting in disordered molecules incorporated within the fibril. The data in **Theory Fig 2**, is the ThT-fluorescence derived kinetics for which we have the largest data sets of (seed) concentrations. Although fewer kinetic traces, we also analyzed the NMR kinetics in the same way (**Theory Figure 3).** Although noisier, the same trend with two distinct regimes and data collapse (when plotted per seed) are indeed noted.

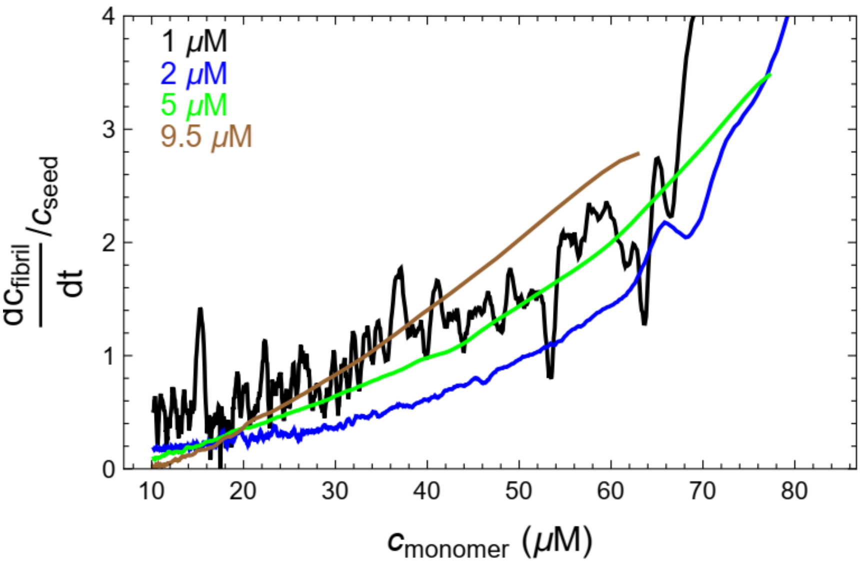

Theory Figure 3. Plot of fibril conversion rate per seed as a function of monomer concentration obtained from the NMR kinetic data (units: hr^{-1}). The curves have been smoothed by averaging over a window of ± 4 adjacent data points.

References

1. J. D. Schmit, Kinetic theory of amyloid fibril templating. *J Chem Phys* **138**, 185102 (2013).
2. J. E. Kohn *et al.*, Random-coil behavior and the dimensions of chemically unfolded proteins. *Proceedings of the National Academy of Sciences of the United States of America* **101**, 12491-12496 (2004).
